# Perforated appendicitis masquerading as a left-sided scrotal abscess: A case report and literature review

**DOI:** 10.1016/j.radcr.2024.09.119

**Published:** 2024-09-26

**Authors:** Elham Zarei, Amir S. Mounesi Sohi, Amir Ghadipasha, Fariba Jahangiri, Ehsan Ranjbar

**Affiliations:** a Department of Radiology, School of medicine, Ali Asghar children hospital, Iran University of Medical Sciences, Tehran, Iran; b Department of Radiology, Firouzabadi Clinical Research Development Unit (FACRDU), Iran University of Medical Sciences, Tehran, Iran; c Department of General Surgery, School of Medicine, Ali Asghar Children hospital, Iran University of Medical Sciences, Tehran, Iran; d Department of Urology, School of medicine, Tehran University of Medical Sciences, Tehran, Iran

**Keywords:** Perforated appendicitis, Scrotal abscess, Patent process vaginalis, Ultrasound

## Abstract

This case report and literature review examine a rare presentation of perforated appendicitis manifesting as a left-sided scrotal abscess in a 3.5-year-old boy. The patient presented with left scrotal swelling and pain, along with a history of abdominal pain and fever. Ultrasound evaluations identified an enlarged, hyper vascular left epididymis and a pelvic abscess, indicative of perforated appendicitis. Laboratory results showed significant inflammatory markers. An urgent appendectomy and surgical exploration of the left hemiscrotum confirmed a gangrenous, perforated appendix in addition to a left-sided scrotal abscess and a patent process vaginalis (PPV). This case highlights the diagnostic complexities in differentiating between genitourinary and gastrointestinal pathologies in pediatric patients. In conclusion and after review of similar cases the role of a patent process vaginalis in infection spread should be underscored and the importance of comprehensive imaging and prompt surgical intervention to mitigate complications must be emphasized.

## Introduction

The testes begin developing in the abdominal cavity around the ninth week of gestation and descend into the scrotum through the process vaginalis, which acts as a communication channel between the peritoneum and the scrotum until it eventually closes (1). It is estimated that a patent process vaginalis (PPV) is existent in 80%-95% of the newborn boys, decreases to 60% by 1 year of age, 40% by 2 years, and persists into adulthood in 15%-37% of males. Abscess formation is a known complication of perforated appendicitis, typically occurring within the abdominal cavity. However, in rare instances, a scrotal abscess can develop following a perforated appendicitis, especially when a PPV is present, facilitating the movement of intraperitoneal pus into the scrotum through the inguinal canal [1]. We depict the case of a 3.5-year-old boy who was admitted to Ali Asghar Hospital with a primary complaint of left scrotal pain and swelling. The child had a history of abdominal pain and fever for 1 week prior to admission. This case highlights the diagnostic challenges and importance of considering atypical presentations in pediatric patients, particularly when distinguishing between genitourinary and gastrointestinal pathologies.

### Case presentation

The patient presented to the emergency department with complaints of left scrotal pain and swelling that had commenced the previous day. On examination, the child was notably restless and exhibited a fever of 38.3°C, while other vital signs were normal. Physical examination of the left hemiscrotum revealed mild swelling and tenderness. Abdominal examination disclosed tenderness in the hypogastric region.

**Fig. 1:**
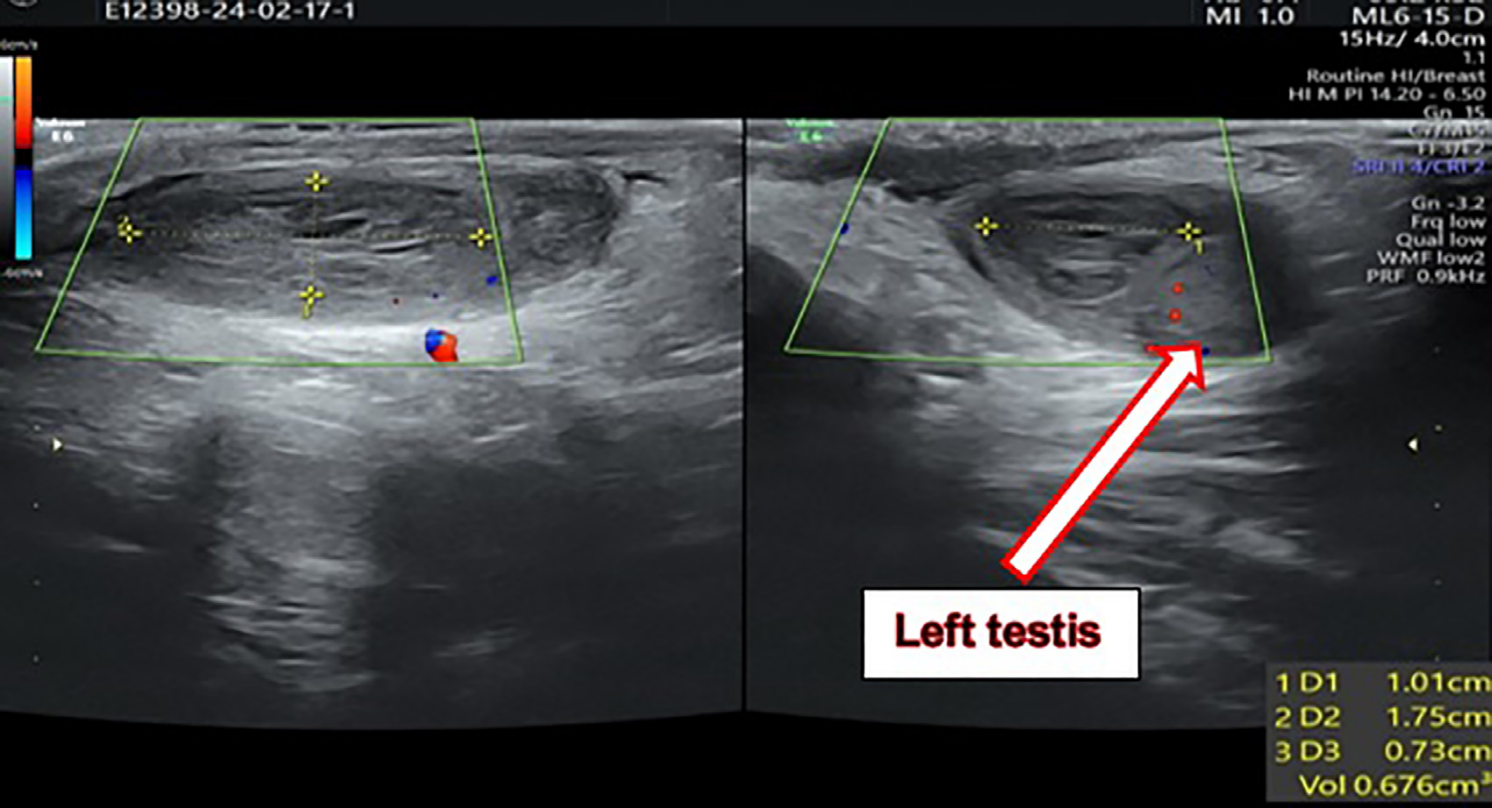
Extra-testicular collection measured about 0.7 cc, adjacent to the upper pole of the left testis.

**Fig. 2:**
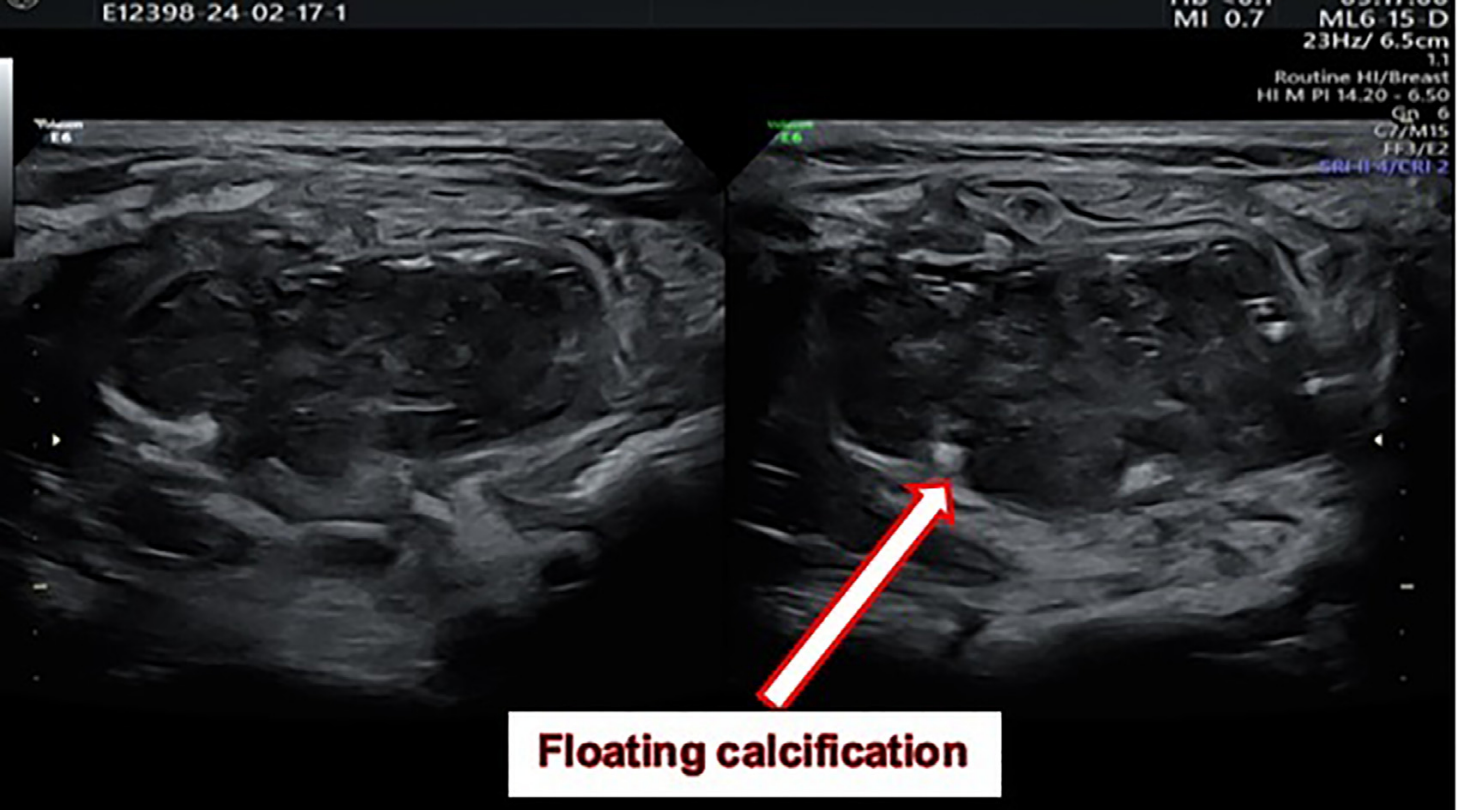
A collection of approximately 20 cc in the pelvis, containing a floating calcification.

Given the symptoms, a scrotal ultrasound was performed to evaluate for acute scrotum. The ultrasound revealed an enlarged and hyper vascular left epididymis with a 0.7 cc extra-testicular collection adjacent to the upper pole of the left testis, suggestive of an inflammatory or infectious process extending into the scrotum (Fig. 1). Due to the patient's history of abdominal pain and the clinical findings, an abdominal ultrasound was subsequently performed. This imaging revealed a collection of approximately 20 cc in the pelvis, posterior to the bladder, containing a floating calcification, raising suspicion for a complicated perforated appendicitis (Fig. 2). Furthermore, increased regional fat echogenicity and mild free echogenic fluid in the pelvis and RLQ were observed in addition to some small reactive mesenteric lymph nodes of short axis diameter of up to 5 mm.

Initial laboratory tests conducted showed significant abnormalities: elevated WBC count (18.3 × 10^3^/mm^3^, normal range: 4-12 × 10^3^/mm^3^), low Hb (10 g/dL, normal range: 11.5-14 g/dL), high platelet count (862 × 1000/mm^3^, normal range: 150-450 × 1000/mm^3^), elevated ESR at 79 mm/hr (normal range: ≤ 10 mm/hr), and elevated CRP at 60.9 mg/L (normal range: 0-10 mg/L). These laboratory findings were indicative of a severe inflammatory response, likely secondary to an infectious process.

The patient underwent an urgent appendectomy and surgical exploration of the left hemiscrotum. Intraoperatively, the appendix was found to be severely inflamed and gangrenous with perforation. The surrounding area was thoroughly debrided to remove infected material. The pathology report detailed the macroscopic and microscopic findings from the appendectomy specimen. Macroscopically, the specimen measured 5 cm in length and 1.5 cm in greatest dimension, with areas of brown discoloration. The lumen was filled with fecaloid material. Microscopically, the appendix exhibited acute gangrenous (perforated) appendicitis with severe peritonitis. In addition, the left testis was also enlarged showing evidences of substantial inflammation and an adjacent collection formation. The process vaginalis was also revealed to be patent in the explorative surgery of the left hemiscrotum.

## Discussion

The case of a 3.5-year-old boy with left scrotal swelling and pain, who was ultimately diagnosed with a perforated appendicitis, underscores the diagnostic challenges and clinical overlap between genitourinary and gastrointestinal pathologies in pediatric patients. This discussion will review the complexities of such atypical presentations and compare our findings with those reported in the literature, drawing insights for clinical practice.

Appendicitis remains one of the most common surgical emergencies in the pediatric population. However, the presentation can be atypical, particularly in younger children [2]. In our case, the primary complaint of left scrotal pain and swelling, combined with a history of abdominal pain and fever, initially pointed towards a genitourinary pathology​​. The ultrasound findings of an enlarged and hyper vascular left epididymis with an extra-testicular collection were consistent with an inflammatory process, which is often misinterpreted as epididymitis or orchitis​.

The subsequent abdominal ultrasound revealing a pelvic collection with a floating calcification redirected the diagnostic consideration towards a gastrointestinal etiology. This underscores the significance of a thorough clinical evaluation and the necessity for high suspicion of gastrointestinal causes in pediatric patients presenting with scrotal symptoms.

A review of the literature highlights several similar cases where appendicitis presented with atypical symptoms, such as scrotal pain and swelling, due to a patent process vaginalis. In a study by Yasumoto et al. [3] from Japan, a 10-year-old boy with left scrotal abscess was found to have a perforated appendicitis. Similarly, Mansoor et al. [4] in Saudi Arabia reported a clinical case of a 4-year-old with a right scrotal abscess due to acute appendicitis. Thakur et al. [5] described 2 cases in the USA where scrotal abscesses developed postappendectomy. This further illustrates the potential for appendicitis to present with scrotal symptoms both pre and postoperatively. The existence of a patent process vaginalis appears to facilitate the spread of infection from the abdominal cavity to the scrotum.

Lee et al. [6] in Taiwan reported a clinical case of a 19-year-old male who developed a right scrotal abscess as a result of retrocecal appendicitis. The patient underwent appendectomy and scrotal exploration, highlighting the need for thorough surgical intervention in such cases. Another case reported by DeFoor et al. [7] in the USA involved an 11-year-old with left scrotal abscess following laparoscopic appendectomy, which underscores the potential complications postsurgery. Bingol-Kologlu et al. from Turkey reported multiple cases where scrotal and inguinal suppuration followed appendectomy. These cases ranged from children aged 3-9 years, all presenting with postoperative complications involving scrotal inflammation and abscess formation [8]. The necessity of recognizing and managing patent process vaginalis during appendectomy was emphasized to prevent such complications.

Saleem in Jordan presented 2 clinical cases of scrotal abscess as a result of perforated appendicitis, 1 involving a 10-year-old and another a 4-year-old. Both cases required surgical drainage and ligation of the patent process vaginalis, reinforcing the critical role of timely surgical intervention [9]. Shehzad et al. described a 16-year-old boy in the UK who presented with acute scrotal pain, initially suspected to be testicular torsion. The diagnosis of perforated retrocecal appendicitis was made intraoperatively, demonstrating the importance of considering appendicitis in the differential diagnosis of acute scrotum [10]. Shahrudin unveiled a case of complicated appendicitis in a 3-year-old boy who was revealed to have relentless fever, persistent leukocytosis and a right swollen and painful hemiscrotum until the ninth postappendectomy day, when the patient was taken to the operation room and 10 ml of pus was observed and drained from the tunica vaginalis, while the right testis was normal, hence, rendering the diagnosis of right scrotal abscess due to a complicated appendicitis. [11]

The comprehensive review of these cases from various geographic locations highlights several key aspects; firstly, appendicitis can present with scrotal symptoms, which are often misleading. The variability in presentation underscores the need for a broad differential diagnosis​. Secondly, the use of both scrotal and abdominal ultrasounds is crucial in identifying the source of infection and guiding appropriate surgical intervention​. Lastly, the presence of a patent process vaginalis plays a significant role in the spread of infection from the abdominal cavity to the scrotum. Recognizing and managing this during appendectomy can prevent postoperative complications.

Our case introduces several new aspects to the existing body of literature. Firstly, the patient's primary symptom was left-sided scrotal pain, which is relatively uncommon. Most cases in the literature report right-sided scrotal involvement, consistent with the typical anatomical position of the appendix (Table 1). This case demonstrates that clinicians should maintain a broad differential diagnosis regardless of the side of scrotal involvement. Secondly, the presence of a floating calcification in the pelvic abscess on ultrasound is a unique finding. This calcification likely represents an appendicolith, which is often associated with perforated appendicitis and severe infection. The identification of such a calcification can be a key diagnostic clue pointing towards appendicitis in atypical presentations

**Table 1:** Cases of scrotal abscess following to perforated appendicitis in pediatric patients.

Study	Country of study	Age of case	Side of scrotum affected	Diagnosis	Treatments	Presence of patent processus vaginalis
Yasumoto et al. [3]	Japan	10	Left	Scrotal abscess due to perforated appendicitis	Scrotal exploration, appendectomy	No
Méndez et al. [2]	Spain	8	Right	Nonperforated retrocecal appendicitis with scrotal abscess	Appendectomy, scrotal exploration	Yes
Thakur et al. [5]	USA	9	Right	Scrotal abscess after appendectomy	Appendectomy, scrotal exploration, drainage	Yes
Thakur et al. [5]	USA	7	Right	Scrotal abscess after appendectomy	Appendectomy, scrotal exploration, drainage	Yes
DeFoor et al. [7]	USA	11	Left	Scrotal abscess after laparoscopic appendectomy	Laparoscopic appendectomy, scrotal exploration, drainage	Yes
Lee et al. [6]	Taiwan	19	Right	Scrotal abscess due to retrocecal appendicitis	Appendectomy, scrotal exploration, percutaneous drainage	Yes
Bingol-Kologlu et al. [8]	Turkey	4	Right	Perforated appendicitis with scrotal abscess	Appendectomy, scrotal exploration, drainage	Yes
Bingol-Kologlu et al. [8]	Turkey	7	Right	Perforated appendicitis with scrotal abscess	Appendectomy, scrotal exploration, drainage	Yes
Bingol-Kologlu et al. [8]	Turkey	9	Left	Perforated appendicitis with scrotal abscess	Appendectomy, scrotal exploration, drainage	No
Saleem [9]	Jordan	10	Left	Scrotal abscess due to perforated appendicitis	Appendectomy, scrotal exploration, drainage, ligation of PPV	Yes
Saleem [9]	Jordan	4	Left	Scrotal abscess due to perforated appendicitis	Appendectomy, scrotal exploration, drainage, ligation of PPV	Yes
Mansoor et al. [4]	Saudi Arabia	4	Right	Scrotal abscess due to acute appendicitis	Appendectomy, scrotal exploration	Yes
Shehzad et al. [10]	UK	16	Right	Perforated retrocecal appendicitis with scrotal abscess	Appendectomy, scrotal exploration, drainage	Yes
Kynes et al. [12]	USA	23 months	Right	Scrotal abscess due to perforated appendicitis	Appendectomy, scrotal exploration	Yes
Zheng et al. [13]	China	15	Right	Scrotal abscess due to gangrenous appendicitis	Appendectomy, scrotal exploration, drainage	No
Keyes et al. [1]	USA	11	Left	Scrotal abscess after laparoscopic appendectomy	Laparoscopic appendectomy, scrotal exploration, drainage	No
Gaffley et al. [14]	USA	11	Left	Scrotal abscess due to perforated appendicitis	Appendectomy, scrotal exploration, drainage	Yes

This case reinforces several important clinical insights; When presented with scrotal pain and swelling, especially in pediatric patients, clinicians should consider both genitourinary and gastrointestinal causes. Scrotal ultrasound can help identify inflammation or abscesses, while an abdominal ultrasound can provide additional clues pointing towards a gastrointestinal origin. In cases of diagnostic uncertainty, a combination of imaging techniques is invaluable​. Even with atypical presentations, appendicitis should remain a top consideration, especially in the presence of abdominal pain, fever, and elevated inflammatory markers.

## Conclusion

In conclusion, the presented case highlights the diagnostic complexities and the need for a comprehensive approach when evaluating pediatric patients with scrotal pain and swelling. This clinical case, alongside a literature review, demonstrates that appendicitis can present with atypical symptoms due to the anatomical spread of infection through a patent process vaginalis. Clinicians must remain vigilant and consider appendicitis in their differential diagnosis, even with atypical presentations. This approach ensures timely intervention and reduces the risk of complications associated with delayed diagnosis.

## Ethics approval and consent to participate

The written informed consent was taken from the patient's parents.

## Patient consent

The patient's parents have consented to the submission of the case report to the journal.
